# Those That Travel by Water

**Published:** 1890-06-21

**Authors:** 


### The Doctor's Pulpit.

### THOSE THAT TRAVEL BY WATER.

"Here we are in the nineteenth century as well prepared to face a sea voyage as our great grandfathers were. The *Calais-Douvre* is a failure; the several kinds of swinging apparatus are useless." This is the comforting declaration of Mr. Thomas Dutton, M.D., an experienced and not injudicious ship's physician. Dr. Dutton has written a pamphlet about sea-sickness, and though he modestly disclaims for it the title of a scientific treatise, he has done his best to go quite to the root of the matter in the way of practical treatment. Travellers by water who suffer grievously from sea-sickness will probably not care much whether their malady be due to "an afflux of blood to the spinal cord"; or to a "disturbed relation of the body to surrounding objects"; or to "unstable vaso-motor equilibrium and depressed circulation"; or to "displacement of the abdominal viscera"; or to "modified visual impressions"; or to any other of the thousand and one "scientific" causes which have been suggested by "scientific" persons whose experience of sea-sickness has been gained in physiological laboratories. What they will care for is some reasonable means of curing the malady when it attacks them; or, still better, of preventing it from attacking them at all.

Dr. Dutton is eloquent on the miseries of those who cannot go to sea without being sick. "Sea-sickness," he says, "is something like toothache, which people make a joke of until they have it themselves." He himself is an old sailor; he has been round the world more than once, and has had experience of thousands of cases; yet he has never mastered his own tendency to the malady. He has seen some persons reduced to such terrible straits that they have all but died. An actual death from sea-sickness alone he never saw.

The medical history of *mal de mer* is not altogether without its funny side. "One morning," says Dr. Dutton, "in going my rounds, after having had a dreadful night of it, I went into a young American lady's cabin to inquire after her health. She said she was much better, and 'guessed' she would be all right after she got on deck, but asked me to stay and 'yarn' with her. I stayed about ten minutes, when suddenly an awful feeling came over me. To her request to stay longer, I replied that if I did we should both have to share the same basin"—a proceeding which would have been by no means romantic. A little conversation overheard one evening seems to indicate that a sea voyage is not always the happiest way of spending one's honeymoon: "Darling, are you better?" says first turtle dove. "No, dearest, worse! What is the use of having a doctor on board who cannot cure sea-sickness, darling?" A significant pause! "It is absurd," says first turtle dove again. "But how foolish of *you* not to spend our honeymoon on shore, dearest! I am sure we shall never enjoy it here." Those people who may be unfortunate enough to have taken to artificial teeth should use exceeding circumspection when they go down to the sea in ships. "What a splendid dentist sea-sickness is! I had the whole of my teeth pulled out at one vomit!" said a patient to the doctor one morning. Several dentists have stated that it is by no means an uncommon occurrence for persons to finish a voyage in a practically toothless condition.

Dr. Dutton is inclined to think that sea-sickness is in many cases nothing more than a righteous retribution following hard upon physiological sins. "A young man," he says, "is about to go abroad. He of course must see everyone and everything before leaving. So for a few weeks before his departure he lives a life of thoughtlessness, eats and drinks far more than is necessary, and lands on board suffering from catarrh of the stomach and congestion of the liver, and just in the proper condition to receive a terrible recompense. The consequence is that instead of having an enjoyable and healthful passage he has a most miserable one; and it takes him the whole time to get himself right again. A young lady does not indulge in alcoholic drinks and other high living; but she makes up for it by an extra quantity of cake, sweets, and afternoon tea." The result in her case is similar to what has been stated of the young man. This indictment of travellers by sea will be generally recognised as true to experience. It is highly probable, too, that the young man and the young woman will still continue to indulge in their alcoholic and afternoon tea "good-byes," preferring to endure all the agonies of prospective *mal de mer* rather than to deprive themselves of those truly British consolations.

"Treatment and Cure" very naturally head the longest chapter in the little book, though the word "Prevention" would more appropriately indicate the actual contents of it. Specifics that "act like charms" are best loved of the public, and particularly for such affections as sea-sickness. But such a thing as a specific for this absurd malady does not, Dr. Dutton declares, exist. Being a practical man, and more concerned with the utility than the severe etiquette of his profession, he has tried the various "quack remedies" which have been vaunted from time to time. All these, with peculiar unanimity, have entirely failed in his hands. But with truly laudable generosity for an orthodox practitioner, he does not blame the remedies so much as the state of the stomach, brought about by the alcoholic and afternoon-tea farewells. The doctor remonstrates with his prospective patients in quite a pathetic tone. You come to me, he says in effect, after your leave takings, with your liver congested, and your stomach filled with pints of sour fermenting gastric juice and saliva, mixed with crude, half-digested food of all sorts. How in the name of common sense do you expect eight or ten drops of a feeble drug to have any effect upon the comparative oceans of vile refuse which your stomach contains? As well imagine that the tiniest mountain rivulet will turn the salt water of the Atlantic into fresh. "How often have I laughed," says he, "when some good-natured lady has brought from a sacred bottle some powder—about as much as would cover a sixpence—and with a serious and believing face, has asked me to allow her to give it to the poor creature, for it would relieve her sufferings at once. Down goes the powder, and generally up it comes again, more promptly than it went down."

Prevention, insists Dr. Dutton, is the only rational method of procedure; and in order really to prevent, the treatment must be begun at least a week before starting on a voyage. Prevention consists in living so rationally for a week or two before going on board as to bring the stomach, liver, brain, and other organs into as perfect a state of health as possible. For this purpose an intelligent dietary and rational exercise are indispensable. Certain simple drugs for the stomach, liver, and brain may also be required. The best results have almost invariably followed treatment of this kind. The case of a lady is given who on her two earliest voyages to India was persistently sick almost the whole of the way. Thus thoroughly alarmed, she was willing to try either Dr. Dutton's treatment or anything else, when a third voyage was in prospect. She tried the prevention method, and was not sick during the whole way, except for about two hours at the start. "The voyage," she wrote, "was a pleasant one. I enjoyed my food thoroughly, and am much improved in health." This lady was naturally bilious, and a victim to constipation. With her the prevention treatment was commenced a fortnight before the day of sailing.

Rules for diet during the voyage are given, the principal one of which seems to be that the passenger should avoid the ship's food as much as possible, and provide himself privately with all sorts of digestible articles before starting. Of special enemies to a prosperous time the ship's steward invariably dishes up at least two. One of these is "fat" in infinite variety: fat in the beef, fat in the bacon, fat in the gravies, fat in the soups, fat in everything. The other is a vile infusion misnamed "tea." This the doctor declares to be ordinarily little better than fluid tannin. Provide your own soups, gravies, beef-teas, biscuits, and, above all, your own tea, he seems to say. Never forget also to take one or more teapots with you, and be sure you infuse the tea yourself. "Four minutes' tea," Dr. Dutton swears by; Sir Andrew Clark pins his faith to a five minutes' decoction. Here the inexorable conditions of space bid us pause. We are not without hope, however, that, thanks to Dr. Dutton's experience and practical common sense, our readers will in future have some really useful knowledge of sea-sickness, and, still better, of its prevention.

